# Are circulating endothelial cells the next target for transcriptome-level pathway analysis in ARDS?

**DOI:** 10.1152/ajplung.00353.2022

**Published:** 2023-02-07

**Authors:** Ana C. Costa Monteiro, Michael A. Matthay

**Affiliations:** ^1^Department of Medicine, Division of Pulmonary and Critical Care, University of California, Los Angeles, California, United States; ^2^Cardiovascular Research Institute, Department of Medicine and Anesthesia, University of California, San Francisco, California, United States

**Keywords:** acute respiratory distress syndrome, ARDS, circulating endothelial cells, transcriptomics

## Abstract

Acute respiratory distress syndrome (ARDS) has had no mortality-improving pharmacological intervention despite 50 years of high-caliber research due to its heterogeneity (Huppert LA, Matthay MA, Ware LB. *Semin Respir Crit Care Med* 40: 31–39, 2019). For the field to advance, better definitions for ARDS subgroups that more uniformly respond to therapies are needed (Bos LDJ, Scicluna BP, Ong DSY, Cremer O, van der Poll T, Schultz MJ. *Am J Respir Crit Care Med* 200: 42–50, 2019; Dickson RP, Schultz MJ, T van der P, Schouten LR, Falkowski NR, Luth JE, Sjoding MW, Brown CA, Chanderraj R, Huffnagle GB, Bos LDJ, Biomarker Analysis in Septic ICU Patients (BASIC) Consortium. *Am J Respir Crit Care Med* 201: 555–563, 2020; Sinha P, Calfee CS. *Am J Respir Crit Care Med* 200: 4–6, 2019; Calfee CS, Delucchi K, Parsons PE, Thompson BT, Ware LB, Matthay MA, NHLBI ARDS Network. *Lancet Respir Med* 2: 611–620, 2014; Hendrickson CM, Matthay MA. *Pulm Circ* 8: 1–12, 2018). A plethora of high-quality clinical research has uncovered the next generation of soluble biomarkers that provide the predictive enrichment necessary for trial recruitment; however, plasma-soluble markers do not specify the damaged organ of origin nor do they provide insight into disease mechanisms. In this perspective, we make the case for querying the transcriptome of circulating endothelial cells (CECs), which when shed from vessels after inflammatory insult, become heralds of site-specific inflammatory damage. We review the application of CEC quantification to multiple disease phenotypes (including myocardial infarction, vasculitides, cancer, and ARDS), in each case supporting the association of CEC number with disease severity. We also argue for the utility of single-cell RNA transcriptomics to the understanding of cell-specific contributions to disease pathophysiology and its potential to uncover novel insight on signals contributing to CEC shedding in ARDS.

## Graphical abstract

**Figure:**
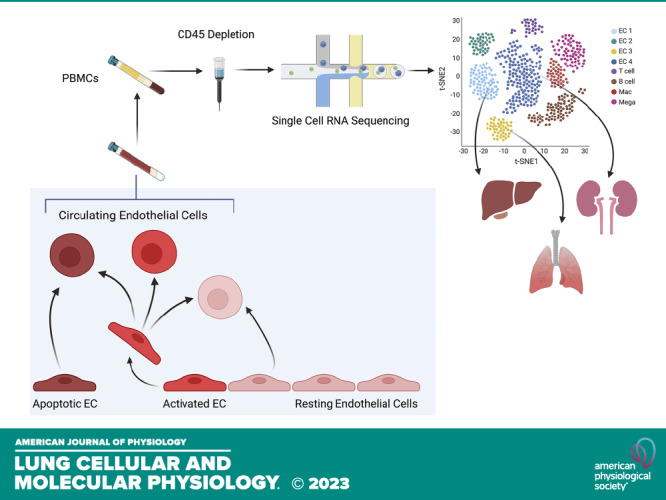

## ADDRESSING ARDS HETEROGENEITY

Several high-quality studies have uncovered plasma biomarkers that encompass epithelial (1, 2, 68) and endothelial injury (3–9), thrombosis (4–8, 10), and inflammation (11–14) and have been proposed as compelling candidates that may aid in pre-selecting a populational subgroup (69, 70) that may best respond to a particular therapy for trial recruitment (predictive enrichment, defined previously) (15). More recently, latent class analyses combining multiple biomarkers have uncovered ARDS subtypes that respond differentially to therapies (14, 16–19). Although these findings represent significant strides towards identifying treatable patient subgroups, a major limitation of biomarker research is an inability to decipher underlying mechanism or ascertaining organ-specific dysfunction, as markers are often shed from various organs and their concentrations in plasma do not reveal the original signaling pathways leading to their release.

Current ARDS research relies on model systems to decipher in vivo mechanisms that may complement observations from clinical trials. For example, the work linking the clinical correlation of plasma elevation of Ang-2 with mortality to the increased lung vascular permeability in mice treated with excess Ang-2 (9, 20, 21) highlighted the central role of vascular injury in the pathogenesis leading to multiorgan dysfunction in ARDS. Likewise, the study of s-RAGE in the pathogenesis of ARDS has relied on both mouse models (22) and patient biomarkers (23) to implicate epithelial damage as a central driver of ARDS outcomes. However, animal models fall short in representing the complexity of human pathophysiology (24, 25), and we continue to have a gap in knowledge between basic experimentation and clinical observation. Importantly, Mendelian randomization and mediation analyses have revealed that genetically regulated elevation of plasma Ang-2 and s-RAGE were associated with ARDS severity (26, 27). A translational approach querying phenotypes of circulatory cells from patient with ARDS may complement findings revealed by causal inference. Specifically, we propose to utilize both bulk and single-cell transcriptomics of CECs to gain mechanistic insight and determine organ-specific contributions to ARDS pathophysiology. This noninvasive approach could provide the keystone linking the signaling cascades deciphered from model systems to the biomarker concentrations observed in patient blood.

## CECs AS MARKERS OF DISEASE SEVERITY

CECs have been described as early as the 1970s (28, 29) as products of vascular shedding that correlate with disease severity in conditions like myocardial infarction, malignancy, and vasculitis (30–34). Their relative quantities are used as markers of disease progression and their elevation implies a central role of vascular injury in the progression of the disease of interest (35). In Mancuso et al. (32), patients with newly diagnosed breast cancer or lymphoma were reported to have a fivefold increase in CECs compared to healthy controls or lymphoma patients in remission. However, the state of CECs and even the mechanisms leading to CEC shedding are still not fully characterized. Zhang et al. (36) proposed that endothelial cells are first activated after insult to either stay tethered or detach into circulation. If released, CECs can further become necrotic or apoptotic. However, Mancuso et al. (32) observed that breast cancer patients had a significant decrease in activated CECs after tumor resection, but at new diagnosis, patients with breast cancer exhibited an increase of both activated and resting CECs, suggesting that activation is either not necessary for endothelial cell untethering or that, once released, CECs can return to a resting state. To clarify this issue, we propose transcriptomic studies of CECs (37) in ARDS to measure the proportion that are activated, necrotic, apoptotic or resting after detachment from the endothelial monolayer.

CECs may not only be casualties of inflammatory injury but may themselves induce inflammatory, procoagulant and immune responses in the endothelium, further contributing to the inflammatory cascade. Kirsch et al. (38) showed that endothelial monolayers induced enhanced leukocyte adhesion in vitro after exposure to CECs from patients with vasculitis as compared to exposure to control CECs. If CECs have the capacity to potentiate inflammation, this may implicate them as registries of important molecular pathways linked to recent injurious signaling. The challenge is to identify effective assays that leverage the information contained in these cells, so that their differential signaling mechanisms, and even their site of origin, can be elucidated.

## ESTABLISHED METHODS IN CEC RESEARCH AND THE ARGUMENT FOR TRANSCRIPTOMICS

The field of CEC research has no established gold standard protocol for identifying CECs from PBMCs. It originally relied on blood immunoprecipitation against the endothelial marker CD-146 followed by electron microscopy, fluorescence immunostaining or direct light microscopy for visualization and enumeration (28, 33, 39, 40). More recently, the identification and enumeration of CECs has relied on multichannel flow cytometry (41–45), which has even enabled identification of activated and resting CECs (32) but there is disagreement on how CECs are phenotypically defined, and there are varying methods for staining and gating the population of interest (43, 46, 47). Duda et al. (48) proposed that optimal CEC gating for flow cytometry was CD31^bright^CD34^dim^CD45^ − ^CD133^−^ and argued that studies utilizing single-epitope immunoprecipitation lacked specificity for CEC quantification. Conversely, proponents of CD-146 immunoprecipitation argue the need for subsequent visualization and staining steps to optimize specificity while highlighting the increased sensitivity of the immunoprecipitation protocol. Indeed, studies utilizing CD146 pull-down reported 10–20 CECs/mL blood in the control arms (42, 47, 48), whereas flow cytometry based studies reported ranges of 10–300 CECs/mL for control groups (32, 44, 49). The debate was somewhat attenuated after a multicenter observational study enrolling 323 patients verified a protocol gating alive and nucleated, CD34^bright^/CD45^−^/CD146^+^ cells as the more reliable phenotype for CEC selection (50), and indeed most studies that utilize flow cytometry to detect CECs from PBMCs do so by a common CD45 depletion step. However, the ongoing variety of protocols utilized in the field (Table 1) argues for further elucidation on the best method to quantify CECs (52, 53). An approach targeting the transcriptomic signature of these cells may allow for better insight into CEC identity and function while improving the specificity and sensitivity of CEC detection. For example, scRNAseq can verify whether CD146 is a sufficiently specific or sensitive epitope for selection of CECs, as it has been utilized in most published CEC isolation protocols to date. Transcriptomic analysis will also define the proportion of CECs that are activated, resting, in apoptosis or necrosis, and enable the identification of altered pathways that may be involved in inflammatory potentiation in ARDS and other critical care disease states.

**Table 1.** ARDS studies that have reported results with circulating endothelial cells

Cases, *n*	Controls, *n*	Method of CEC Quantification/Phenotype	CEC Number in Cases	CEC Number in Controls	Reference
17 Moderate-severe ARDS	9 Mild ARDS, 13 sepsis without ARDS, 13 nonseptic patients, and 12 healthy volunteers	CD146 antibody-based immunomagnetic isolation and UEA1-FITC staining method on *day 1* post-ARDS	27.2 (18.3–49.4) cells/mL	17.4 (11–24.5) cells/mL in mild ARDS; 18.4 (9.1–31) cells/mL in non-ARDS sepsis, 1.8 (0.4–3.7) cells/mL in healthy controls	(45)
19 ICU-level COVID-19 patients	80 Non-ICU, hospitalized COVID-19 patients	CD146 antibody-based immunomagnetic isolation and enumerated using a fluorescence microscope after acridine orange labeling. CECs were identified according to the following criteria: stained rosette cells, size > 15 µm, and bearing more than 5 beads	49 (24–103) cells/mL	18 (6–70) cells/mL	(42)
19 COVID-19 patients with severe infection (defined as requiring >5 L oxygen)	14 Uninfected patients, 14 asymptomatic/mild COVID-19 patients (defined as having no oxygen requirement); 23 moderate COVID-19 patients (defined as requiring 1–5 L oxygen)	Flow cytometry of PBMCs. CECs identified as CD45^−^CD31^+^CD34^+^CD146^+^	0.05% of total PBMCs	0% Total PBMCs in uninfected and mild/asymptomatic disease; 0.05% of total PBMCs in moderate COVID-19 disease	(51)

ARDS, acute respiratory distress syndrome; CECs, circulating endothelial cells.

## THE FUTURE OF CEC RESEARCH IN ARDS

**Figure 1.**
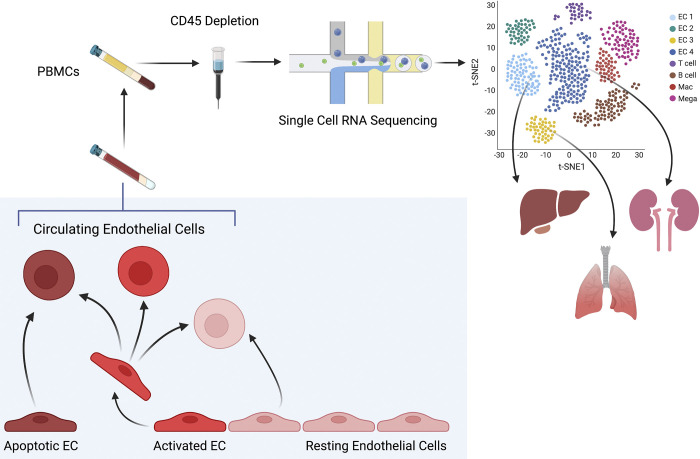
Proposed workflow for isolation of circulating endothelial cells. Endothelial cells (ECs) may be released into circulation after activation and/or initiation of apoptosis/necrosis, and some literature has supported the presence of nonactivated ECs in circulation. The phenotypes of circulating endothelial cells are incompletely characterized. Single-cell RNA sequencing of an enriched PBMC population (we propose CD-45 depletion) may enable the determination of the different states of circulating endothelial cells and potentially identify their organ of origin. We propose the comparison of the transcriptomes of patients with ARDS and non-ARDS. ARDS, acute respiratory distress syndrome. [Image created with BioRender.com and published with permission.]

Single-cell RNA sequencing has been paramount in describing rare cell populations in immunology, epithelial, and endothelial biology (54–58). CECs are an untapped source of transcriptomic information and scRNAseq can provide an exploratory approach that may enable insights into mechanisms leading to endothelial cell damage and untethering. The endothelial transcriptomic signature has been well described in vascular biology (54, 55, 59–61) and provides a solid foundation for the evaluation of the CEC transcriptome. Different groups have reported specific signatures for varied endothelial subtypes, such as those for arterial, venous, and capillary endothelium and have described unique transcriptomic changes after endothelial activation (62, 63). Interestingly, endothelial signatures based on organ-specific location have also been observed (60, 63). Kalucka et al. identified lung-specific endothelial cell signatures by scRNAseq of murine tissue, such as unique immunoregulatory-related signatures, an enhanced Fox1 network of transcription factors, and specific enhancement of genes associated with cAMP metabolism, as well as unique markers, such as Tmem100, a transmembrane protein in the MAPK pathway (59). Findings from Paik et al. (64) support that markers of murine lung endothelial cells are conserved in humans. Schupp et al. further characterized the diversity of human endothelial cells by generating an integrated endothelial cell atlas derived from 6 scRNAseq datasets of human lung (65). We can leverage this information to evaluate the transcriptomic signatures identified by scRNAseq of blood to determine the makeup of CECs from patients with ARDS compared to sepsis or controls (Fig. 1). Endothelial cells become activated in response to insult, triggering leukocyte adhesion and initiating angiogenesis pathways (30, 32, 33, 36). Deciphering the proportion of CECs that are activated in ARDS may provide mechanistic insight into how organ damage is exacerbated after initial insult, and may provide us with clues on pathways to recovery. Likewise, the extent of lung endothelial injury relative to endothelial injury from other organs may be estimated in ARDS compared to control groups. Lessons from such studies may then be translated into clinically applicable assays; for example, if pulmonary CEC numbers were to confidently correlate with a particular plasma-soluble protein, we could use the latter as a verified proxy marker of pulmonary damage.

## THE POTENTIAL AND ANTICIPATED PITFALLS FOR FUTURE CEC RESEARCH IN ARDS

Although there is great value from transcriptomic characterization of CECs, there are several challenges with this approach. First, CECs are rare, in the order of 10–300 cells/mL of whole blood in control subjects, and scRNAseq is limited by the number of cells analyzed. To resolve this, one or more enrichment steps may be required before scRNAseq, such as apheresis or differential centrifugation for PBMC isolation followed by immune enrichment via a depletion step against the leukocyte marker CD45, or by a flow cytometry sorting approach that optimizes sensitivity to CECs at the expense of specificity, such as one limited to CD45^−^ cells, as scRNAseq would further outline phenotypes to ensure specificity of cell populations. Second, the optimal timepoint for harvesting CECs is not known. The lifespan of CECs once released in circulation has not been thoroughly investigated and it is not established whether a peak in CEC numbers would anticipate or lag behind disease severity. Further analysis on the kinetics of CEC numbers in ARDS may be required before a commitment to a set timepoint for scRNAseq and may require several timepoints to adequately evaluate correlation with clinical course. Some of this research has been initiated in patients infected with COVID (51), where CEC numbers increased within 5 days of COVID+ PCR for patients requiring invasive mechanical ventilation; however, this timeline may differ based on ARDS etiology. Interestingly, several studies of CECs in patients after cardiopulmonary exercise testing revealed that CEC numbers increased as shortly as 5 min after exercise, but recovery time to baseline was not reported (53, 66). Lastly, a low signal strength, such as a rare cell population, raises concerns for its clinical relevance. However, in the oncology field, CECs have been documented as important markers of therapeutic response (35, 44), and mitigating CEC elevation has become an important therapeutic goal in vasculitis and cardiovascular disease (39, 67). CECs have the potential to uncover a new avenue of diagnostic and mechanistic discovery without the need for invasive diagnostic approaches. The latest molecular techniques provide a new opportunity to study these cells and decipher signaling that may have relevance in terms of pathogenesis and prognosis in ARDS.

## DISCLOSURES

No conflicts of interest, financial or otherwise, are declared by the authors.

## AUTHOR CONTRIBUTIONS

A.C.C.M. prepared figures; A.C.C.M. drafted manuscript; A.C.C.M. and M.A.M. edited and revised manuscript; A.C.C.M. and M.A.M. approved final version of manuscript.
